# Chlorpromazine-Induced Relapse of Tourette Syndrome in a Patient With Intellectual Disability, Attention Deficit Hyperactivity Disorder, and Schizophrenia

**DOI:** 10.7759/cureus.10732

**Published:** 2020-09-30

**Authors:** Anum Maqsood, Salman Akram, Faisal Akram

**Affiliations:** 1 Internal Medicine, Howard University Hospital, Washington, DC, USA; 2 Internal Medicine, Rawalpindi Medical University, Gujranwala, PAK; 3 Psychiatry, Department of Behavioral Health, Saint Elizabeths Hospital, Washington, DC, USA

**Keywords:** tourette syndrome, chlorpromazine, attention deficit hyperactivity disorder, schizophrenia

## Abstract

Tourette syndrome (TS) is a chronic neuropsychiatric disorder characterized by recurrent multiple motor and vocal tics that last for at least one year and follow a waxing and waning course. A fundamental step in the pathophysiology of TS is the hyperactivity of dopaminergic system leading to increased dopamine release in the cortical-basal ganglia-thalamo-cortical (CBGTC) circuits, thereby providing the rationale for treatment with dopamine receptor, in particular D2, antagonists. Although antipsychotics have shown considerable efficacy against tics in most patients, there have been cases of paradoxical onset of tics in individuals without history, and relapse or exacerbation of tics in individuals with a history of tic disorders upon initiation of antipsychotics. Here we report a case of an individual with intellectual disability, attention deficit hyperactivity disorder (ADHD), and schizophrenia, who experienced a relapse of TS symptoms after initiation of chlorpromazine therapy.

## Introduction

Tourette syndrome (TS) is a neuropsychiatric disorder characterized by childhood onset of chronic motor and vocal tics with “a waxing and waning” course [1,2]. It is a lifelong disorder with the most severe symptoms occurring between 8 and 12 years, usually followed by a gradual decline in severity [3]. Tics are defined as sudden, rapid, recurrent, and non-rhythmic movements or vocalizations with varying degree of purposefulness and voluntariness [2]. TS has a strong genetic predisposition with obsessive-compulsive disorder (OCD) and attention deficit hyperactivity disorder (ADHD) [4]. Other comorbid psychiatric disorders reported to be seen in patients with TS include autism spectrum disorder and bipolar disorder. Tics are typically exacerbated by stress, anxiety, and fatigue and often preceded by a premonitory sensation or urge. Here we present a case of an individual with intellectual disability, ADHD, and schizophrenia, who experienced a relapse of TS symptoms after the initiation of chlorpromazine therapy.

## Case presentation

A 26-year-old male patient presented to our department with auditory hallucinations, mutism, posturing, disorganized speech, and aggressive behavior. His mental health problems started at age three years with poor social responsiveness and emotional dysregulation, and gradually progressed to include impulsivity, anger outbursts, hyperactivity, vocal and motor tics, and cognitive difficulties. Subsequently, he was diagnosed with mild intellectual disability, ADHD, and TS during early childhood. At age 16 years, he started experiencing auditory hallucinations and disorganization of thought process (loose associations and thought blocking) that led to the diagnosis of schizophrenia. A review of medical records showed that he had been taking atomoxetine for ADHD and TS until age 16 years, which was discontinued upon the onset of psychotic symptoms. His psychotic and affective symptoms were poorly managed by risperidone and valproic acid over the last six years, primarily due to the high degree of medication non-compliance. On initial evaluation at our department, the patient did not have signs or symptoms of TS and had not experienced motor or vocal tics for many years. After a thorough medical workup, treatment for an acute psychotic episode of schizophrenia was initiated with risperidone and valproic acid. Chlorpromazine was added to the medication regimen to control recurrent aggression and violence. Roughly two weeks after the initiation of chlorpromazine, the patient started exhibiting involuntary, repetitive, and non-rhythmic myoclonias-like movements of the right upper limb with high amplitude and velocity, as well as grunting, sniffing, throat clearing, and eye blinking. Coprolalia was also observed. Routine biochemical investigations including liver function tests, serum calcium, phosphorous, T4, and electrolytes were normal. Urinalysis and hemogram were normal. Valproic acid levels were initially in the higher range and normalized after dose reduction, with no effect on vocal or motor tics. Electrocardiogram (EKG) was normal. MRI of the brain was unremarkable. A two-hour video electroencephalogram (EEG) showed non-specific abnormalities. Based on clinical presentation and subsequent workup, a diagnosis of acute relapse of Tourette’s disorder was made. Because of inadequate response and worsening motor tics, chlorpromazine was tapered till discontinuation, and clozapine therapy was initiated. Discontinuation of chlorpromazine and initiation of clozapine therapy caused a gradual decrease in frequency and amplitude of vocal and motor tics over a period of one year. He was transferred from our service after he achieved partial remission of psychotic symptoms and was essentially free of vocal and motor tics.

## Discussion

The main finding of this case report is the re-emergence of vocal and motor tics in a patient with a history of TS, soon after initiation of chlorpromazine therapy. Although it may seem paradoxical as antipsychotics are the first-line treatment for TS, several cases of antipsychotic-induced tics have been previously reported [5-10]. One systematic review by Kim et al. identified 60 cases of antipsychotic-induced tics, with 30 cases associated with first-generation antipsychotics and 27 with second-generation antipsychotics [5]. In addition, the occurrence of vocal tics was significantly higher in the first-generation antipsychotic use as compared to second-generation antipsychotics. Most of the cases have been reported in the context of tardive syndromes and tics appeared after long-term antipsychotic therapy. However, patients with a history of inattention, hyperactivity, and motor and vocal tics experienced relapse or exacerbation of tics relatively earlier during the course of antipsychotic therapy [5]. For example, Gualtieri and Patterson reported two cases, both aged nine years, with symptoms of inattention, hyperactivity, and aggression, who developed phonic and motor tics two weeks after initiation of haloperidol and thioridazine, respectively [9]. Similarly, Huang et al. reported the recurrence of motor tics five days after initiation of aripiprazole in a 30-year-old male with a history of chronic motor tics [10]. These reports are in corroboration with Cianchetti et al., who reported three cases of TS that did not show any improvement with several neuroleptic drugs at appropriate dosages but improved significantly on dopamine agonist, pergolide [11], raising a possibility that a subset of individuals with TS may have different pathways that lead to vocal and motor tics. This is further supported by evidence of the efficacy of treatment with dopamine agonists in TS in some, but not all, open-label [12] and randomized controlled trials [13,14].

Over the past two decades, there have been several advances in our understanding of the neurodevelopmental origins and underlying neurobiology of TS [15]. Current evidence points to dysfunction in cortical-basal ganglia-thalamo-cortical (CBGTC) circuits leading to abnormal inhibition of undesired actions. In particular, the dopamine hypothesis proposes a hyperresponsive spike-dependent dopaminergic system leading to abnormal phasic dopamine bursts, as evidenced by (1) increased dopamine release in the putamen, medial frontal gyri, and anterior cingulate cortex; (2) increased ventral striatal monoaminergic innervation; and (3) increased dopamine transporter binding in the striatum [15-17]. In addition, abnormalities in glutamatergic and cholinergic pathways regulating the dopaminergic system have also been reported [18,19]. Interestingly, one theory about the pathogenesis of tardive dyskinesia, including tardive Tourettism, is that long-term antipsychotic therapy leads to D2 receptor upregulation with postsynaptic dopamine receptor hypersensitivity [20]. Considering that onset of tic relapse in our case and many others with a childhood history of tics was relatively earlier, one plausible explanation of early-onset antipsychotic-induced tic disorder may be that, at least in a subset of TS patients, antipsychotics act on an already existing hypersensitive dopaminergic system, leading to increased phasic (burst firing) dopamine release. The specificity of antipsychotics-induced tics to phasic dopamine release is further supported by the fact that tics resolved after discontinuation of antipsychotic drugs in most case reports, unlike tardive dyskinesias, which typically persist after discontinuation of antipsychotic drugs [5].

## Conclusions

This case report, along with previous case reports, contributes to the knowledge base that antipsychotics may paradoxically cause relapse or exacerbation of tics. More research is needed to explore if there exists a subset of individuals with tic disorders who are particularly vulnerable to this phenomenon, which may have treatment implications such as therapy with dopamine agonists or drugs with less dopamine blockade.
